# Commentary on: Antibody and B Cell Responses to Plasmodium Sporozoites

**DOI:** 10.3389/fimmu.2015.00113

**Published:** 2015-03-17

**Authors:** Jerome P. Vanderberg

**Affiliations:** ^1^Department of Microbiology, New York University School of Medicine, New York, NY, USA

**Keywords:** malaria, plasmodium, sporozoite, immunity, antibodies, blood, liver, mosquitoes

Dups et al. (1) assess how antibody and B cell responses to *Plasmodium* sporozoites may function as protective immune responses. They cover immune induction quite extensively but discuss virtually nothing on effector mechanisms of antibody responses against sporozoites. Because their review should be balanced by reference to studies describing effector mechanisms, this commentary will confine itself to recounting some of these.

*In vitro* exposure to serum from immunized mice results in an antibody-mediated precipitant projecting from sporozoites (2). Because of the striking way in which immune serum deformed sporozoites, this “CSP” reaction was postulated to be the basis for a humoral component of immunity against sporozoites. *In vitro* inhibition of sporozoite motility by serum from immune animals subsequently suggested that anti-sporozoite antibodies can function by blocking sporozoite motility (3). Further *in vitro* studies showed that monoclonal antibodies against the CS antigen blocked *in vitro* invasion by sporozoites into target cells (4, 5). Subsequent intravital studies revealed *in vivo* inhibition of sporozoite motility and inhibition of sporozoite invasion of dermal blood vessels by antibodies (6). And an association between high levels of CS antibodies and protection was shown in the first successful human immunization with radiation-attenuated sporozoites (7).

Anti-sporozoite antibodies act even earlier during challenge by mosquito bite, by inhibiting release of sporozoites from the mosquito proboscis into skin (8). This appears to be the initial manifestation of functional immunity against pre-erythrocytic malaria parasites. Analysis of kinetics of *Plasmodium berghei* sporozoites injected by mosquitoes into sporozoite-immunized vs. non-immunized mice showed significantly fewer sporozoites were deposited in immune mice (8). CS protein is released by sporozoites into media *in vitro* (9) and into saliva within salivary glands of infected mosquitoes (10, 11). Thus, CS protein released into mosquito saliva together with sporozoites is injected into hosts while mosquitoes probe for blood meals. This mosquito-injected CS protein appears to interact with homologous anti-CSP antibodies within immunized hosts at sites of saliva release; the interaction produces immune complexes that interfere with free release of sporozoites by mosquitoes *in vitro* and *in vivo* (8).

Some sporozoites injected by mosquitoes into immunized hosts may successfully escape into the blood. Indeed, sporozoites that bypass skin by being injected intravenously by syringe into mice passively immunized with immune serum are cleared effectively from the circulation (12), indicating a functional role for antibodies against “break-through” sporozoites that have reached the blood.

Conceivably, antibody-coated sporozoites may be blocked during attempted passage through Kupffer cells prior to hepatocyte invasion (13, 14). *In vitro* studies have documented that opsonized sporozoites are prone to phagocytosis (15–17), suggesting that Kupffer cells can eliminate opsonized sporozoites *in vivo*. Furthermore, antibodies also inhibit invasion and traversal through hepatocytes, events that rely on sporozoite motility (18).

Thus, anti-sporozoite antibodies can act sequentially against sporozoites at different stages of sporozoite invasion from the skin to invasion of dermal blood vessels, to passage from Kupffer cells into hepatocytes, and subsequent traversal of sporozoites through a series of hepatocytes.

## Conflict of Interest Statement

The author declares that the research was conducted in the absence of any commercial or financial relationships that could be construed as a potential conflict of interest.
